# Cognitive behavioral therapy for insomnia as a suicide prevention strategy: a protocol for a systematic review and meta-analysis

**DOI:** 10.1093/sleepadvances/zpaf070

**Published:** 2025-10-09

**Authors:** Cagdas Türkmen, Carlotta L Schneider, Yuki Furukawa, Jens H van Dalfsen, William V McCall, Wilfred R Pigeon, Andrew S Tubbs, Michael L Perlis, Dieter Riemann, Kai Spiegelhalder, Knut Langsrud, Håvard Kallestad, Elisabeth Hertenstein

**Affiliations:** Department of Addictive Behaviour and Addiction Medicine, Central Institute of Mental Health, Medical Faculty Mannheim, University of Heidelberg, Mannheim, Germany; Faculty of Medicine, Department of Psychiatry, University of Geneva, Geneva, Switzerland; Technical University of Munich, TUM School of Medicine and Health, Department of Psychiatry and Psychotherapy, Munich, Germany; Department of Neuropsychiatry, University of Tokyo Hospital, Tokyo, Japan; Department of Psychiatry, University Medical Center Groningen, Groningen, The Netherlands; Department of Psychiatry and Health Behavior, Medical College of Georgia, Augusta University, Augusta, GA, United States; Department of Psychiatry, University of Rochester Medical Center, Rochester, NY, United States; Center of Excellence for Suicide Prevention, U.S. Department of Veterans Affairs, Canandaigua, NY, United States; Department of Psychiatry, Washington University School of Medicine, St. Louis, MO, United States; Behavioral Sleep Medicine Program, Department of Psychiatry, The School of Nursing, University of Pennsylvania, Philadelphia, PA, United States; Department of Psychiatry and Psychotherapy, Medical Center – University of Freiburg, Faculty of Medicine, University of Freiburg, Germany; Center for Basics in NeuroModulation (NeuroModulBasics), Faculty of Medicine, University of Freiburg, Freiburg, Germany; Department of Psychiatry and Psychotherapy, Medical Center – University of Freiburg, Faculty of Medicine, University of Freiburg, Germany; Østmarka Psychiatric Department, St. Olavs Hospital PH, Trondheim, Norway; Department for Research and Development, St. Olavs Hospital PH, Trondheim, Norway; Department of Mental Health, Norwegian University of Science and Technology, Trondheim, Norway; Faculty of Medicine, Department of Psychiatry, University of Geneva, Geneva, Switzerland

**Keywords:** insomnia, sleep–wake disorder, suicidality, suicide, cognitive behavioral therapy, CBT-I

## Abstract

Insomnia is a prevalent and debilitating sleep–wake disorder, with growing evidence indicating that it is an independent risk factor for suicidal ideation and behaviors. Cognitive behavioral therapy for insomnia is the recommended first-line treatment for chronic insomnia. However, its effect on suicidal ideation and behaviors in those with insomnia has not been well-characterized. Thus, the aim of the planned meta-analysis is to quantify the effects of cognitive behavioral therapy for insomnia on suicidal ideation, suicidal behaviors, and suicide deaths in adults with insomnia with and without comorbidities. This study will include randomized controlled trials comparing cognitive behavioral therapy for insomnia with a control condition with no presumed strong effect on insomnia in individuals with insomnia according to standardized diagnostic criteria or a clinically relevant screening score. Both published and unpublished randomized controlled trials will be retrieved through a systematic search in major databases and trial registries. The primary outcomes include suicidal ideation, suicidal behaviors, and suicide deaths, assessed post-treatment and at follow-ups. We will only consider randomized controlled trials reporting suicide-related outcomes and/or enrolling participants with suicidal ideation or behaviors. For continuous data, a random-effects meta-analysis will be conducted to estimate (standardized) mean differences. In the case of categorical data, a random-effects logistic regression meta-analysis model will be used. The risk of bias of the primary outcomes will be evaluated using the Cochrane Risk of Bias 2 tool. The certainty of evidence will be assessed using the Grading of Recommendations, Assessment, Development and Evaluation framework.

**Systematic review registration**: PROSPERO-ID CRD420250628820.

Statement of Significance Insomnia is an important risk factor for suicidal thoughts and suicide attempts. Understanding the extent to which treating insomnia may reduce suicide risk is thus crucial for prevention. This meta-analysis will evaluate how effective cognitive behavioral therapy for insomnia is in reducing suicidal thoughts, behaviors, and deaths. The results of this study could help to improve suicide prevention strategies, inform clinical practice, and guide future research on targeting sleep to reduce suicide risk.

## Introduction

Insomnia is a highly prevalent and debilitating sleep–wake disorder, affecting approximately 1 in 10 individuals in the general population [1]. It is associated with reduced health-related quality of life [2], impaired work productivity [3] and an increased risk of developing psychiatric disorders [4]. Accumulating evidence indicates that insomnia is also a significant risk factor for suicidal ideation and behaviors across various age groups [5–10]. Thus, the effective treatment of insomnia is of high public health importance.

Cognitive behavioral therapy for insomnia (CBT-I) is the recommended first-line treatment for chronic insomnia among adults [11]. It is a multicomponent treatment including sleep hygiene education, relaxation strategies, cognitive restructuring, as well as sleep restriction and stimulus control as the core behavioral components. The aim of stimulus control is to strengthen the association between the bed and sleep, while sleep restriction involves restricting the time spent in bed to increase sleep efficiency. Recent meta-analyses have shown that CBT-I is effective for the treatment of insomnia in individuals with comorbid mental health and alcohol use disorders [12–14], where insomnia can exacerbate symptoms and increase the risk of relapse [15, 16]. While CBT-I may reduce comorbid symptom severity [12], there is a paucity of evidence synthesis addressing whether CBT-I may also reduce suicidal ideation and behaviors.

Since 2010, there has been a call to action to evaluate sleep interventions as a strategy for suicide prevention [17]. There has been a slow but steady response to this call, with initial observational studies suggesting that CBT-I may reduce suicidal ideation [18, 19]. Importantly, a recent randomized controlled trial (RCT) has found that insomnia remission may be a key mechanism through which CBT-I alleviates and prevents suicidal ideation [20]. Given recent meta-analytic evidence indicating that CBT-I is more effective than pharmacotherapy in achieving short- and long-term insomnia remission [21], CBT-I may be particularly well-suited for suicide prevention. One potential mechanistic pathway underlying the relationship between insomnia and suicidal phenomena is hyperarousal [22–24], which CBT-I has been hypothesized to reduce [25, 26].

Research on CBT-I as a suicide prevention strategy has recently gained significant momentum, particularly through RCTs contributing to the evidence base [20, 27–29]. However, access to and scalability of CBT-I remain limited due to a paucity of trained providers. Digital formats of CBT-I have emerged as viable alternatives to face-to-face delivery [30], with recent research showing promising results for digital CBT-I in reducing the risk of suicidal ideation [20]. This suggests that CBT-I not only holds promise for suicide prevention but also holds potential for greater scalability and accessibility, which could further strengthen suicide prevention efforts.

Despite these promising developments, uncertainty remains regarding the overall effect of CBT-I on suicidal ideation and behaviors. A recent meta-analysis reported a modest effect of sleep interventions in reducing suicidal ideation. However, these findings were limited by substantial heterogeneity among the relatively few included studies, which may be due to the broad inclusion of various sleep disorders (e.g. nightmares, hypersomnia, insomnia), as well as a mix of pharmacological and behavioral interventions and different age groups (youths and adults) [31]. A preliminary search has also identified relevant newly published trials [32, 33] and an unpublished, completed trial [34] for CBT-I. Building on these recent advances, the planned meta-analysis aims to update the current evidence by incorporating new publications and unpublished data, while narrowing the focus to CBT-I. Specifically, we aim to quantify the effects of CBT-I, compared with control conditions, on suicidal ideation, suicidal behaviors, and suicide deaths among adults with insomnia with and without comorbidities.

## Methods

The information in this protocol is reported in accordance with the guidelines of the Preferred Reporting Items for Systematic Review and Meta-Analysis Protocols (PRISMA-P) [35, 36]. The completed PRISMA-P checklist is provided in Supplement 1. The study was prospectively registered on PROSPERO (CRD420250628820). In the event of amendments to the study protocol, each amendment will include the date, a description of the change, and the rationale.

### Selection criteria

We will include published and unpublished records reporting on RCTs that compare CBT-I in any delivery format to control conditions with no presumed strong effect on insomnia (i.e. placebo, sleep hygiene, self-help, waitlist, no treatment) in parallel-group designs. CBT-I must include stimulus control or sleep restriction as behavioral components, in accordance with findings from a recent network meta-analysis identifying these components as essential for achieving insomnia remission [37]. CBT-I may be combined with other treatments (e.g. specific to a psychiatric disorder), provided that the control group receives the same treatment. We will consider adults (≥18 years) of any gender with insomnia with or without comorbidity according to diagnostic criteria (i.e. the Diagnostic and Statistical Manual of Mental Disorders [38, 39], the International Classification of Sleep Disorders [40] or Diseases [41, 42]), or a validated, clinically meaningful screening score. We will only consider studies reporting suicide-related outcomes and/or enrolling participants with suicidal ideation or behaviors. Disagreements regarding study selection will be resolved by consensus or, if necessary, with the involvement of another member of the review team.

### Search strategy

The following databases and registers were systematically searched to identify records published from inception through March 21, 2025: Medline, Embase, Cochrane Library, PsycInfo, and ClinicalTrials.gov. No language restrictions were applied. An RCT filter was applied to all databases except the Cochrane Library. The search will be complemented by screening the reference lists of the included studies and of relevant systematic reviews on this topic [31, 43]. Additionally, forward citation searches (i.e. identifying studies that have cited the included studies and relevant systematic reviews on this topic) will be performed to capture any further eligible records. Search strings and the number of hits for each database/register are provided in Supplement 2.

### Primary and secondary outcomes

The primary outcomes include suicidal ideation, suicidal behaviors, and suicide deaths, assessed post-treatment and at follow-up(s). The secondary outcomes include insomnia severity, also measured post-treatment and at follow-up(s), as well as depression severity and hopelessness, which are key correlates of suicidal ideation, behaviors, and suicide deaths [44].

Regarding the primary outcomes, suicidal ideation refers to thoughts of self-harm with the intent to die and includes self-reported or clinician-rated thoughts of suicide, assessed via clinical records, structured interviews, or validated instruments (e.g. Beck Scale for Suicide Ideation [45], Columbia-Suicide Severity Rating Scale [46]). Suicidal behaviors encompass any reported suicide attempts or preparatory behaviors, based on clinical records, structured interviews, or validated instruments (e.g. Columbia-Suicide Severity Rating Scale [46]). Suicide deaths, typically verified through medical records or death registries, will be included when reported. For the secondary outcomes, all assessments should be conducted using validated instruments (e.g. Insomnia Severity Index [47] for insomnia severity).

Based on preliminary searches, suicidal ideation and the secondary outcomes are commonly measured using continuous outcome measures. Thus, continuous data will be prioritized over categorical data in the analyses for these outcomes. Suicidal behaviors and suicide deaths are typically measured as categorical variables, and therefore, categorical data will be prioritized for these outcomes.

### Record management and screening

Records will first be imported into EndNote (Clarivate, Version 21, 2023) [48]. and deduplicated using the software’s built-in functions. The deduplicated records will then be exported from EndNote to an Excel spreadsheet for formal screening.

Two reviewers will independently screen the titles and abstracts of all studies and assess whether studies are thematically relevant to the relationship of interest. At this stage, essential eligibility criteria, for which information is typically available in abstracts, will be checked, including study design (RCT), age group (adults), intervention type (CBT-I), and type of control group. Records will initially be coded as clearly irrelevant (excluded prior to full-text review) or potentially relevant (proceeding to full-text review).

At the full-text review stage, potentially relevant records will be comprehensively assessed to ensure that all inclusion/exclusion criteria listed under *Selection criteria* are met. We will also verify that outcome data are available in the correct format for analysis and ensure that no duplicate studies are included. For unpublished studies, we will also report whether the study is ongoing and note the estimated completion date to determine the feasibility of inclusion in the meta-analysis. Finally, records will be coded as either included or excluded, with reasons documented for each exclusion.

### Data extraction

Two reviewers will independently extract data from all included trials for the following variables: authors, year of publication, number of participants in the CBT-I/control groups at each timepoint, age of participants at baseline, percentage of women at baseline, race/ethnicity, number of follow-up assessments, time between baseline and each follow-up, criteria for insomnia, outcome measures, CBT-I components, number of CBT-I sessions, treatment duration, and details regarding control conditions. This information will be summarized narratively in the text and/or presented in tabular form.

Outcome data will be extracted independently by two reviewers using a pre-defined Excel sheet for the meta-analyses. Depending on the frequency of continuous versus categorical data reporting, we may opt to harmonize the data to enhance statistical power and reduce the number of analyses by reaching out to study authors. For instance, if most studies reported continuous data and it is feasible to obtain continuous data from studies that reported categorical data (e.g. those that transformed continuous data into binary outcomes using cut-off scores), we will request these data from the principal investigators.

Principal investigators of both unpublished and published studies will be contacted up to three times, with a 2-week interval between each attempt, to request unpublished data or address other relevant inquiries.

### Statistical analysis

For pairwise comparisons informed by ≥3 RCTs, we will perform a meta-analysis if the RCTs are sufficiently homogeneous with respect to design and comparator. For continuous data, mean differences (MDs) or standardized mean differences (SMDs), along with 95% confidence intervals (CIs), between the CBT-I group versus control groups will be calculated for the outcomes post-treatment and at follow-up(s). MDs will be used when all studies measured an outcome on the same scale, while SMDs will be used when multiple scales are used across studies. In the case of SMDs, Hedges’ g will be used as a measure of effect size. We will rely on post- and (follow-up-) means, instead of a change score between baseline and post- (and follow-up-) means. Based on the assumption that there is a distribution of true effect sizes rather than a single true effect size, a random-effects model will be used when pooling the primary studies, in line with the recommendation by Borenstein *et al.* (2010) [49]. Forest plots will be presented to visualize the results. The degree of heterogeneity between studies will be assessed using I^2^, which describes the proportion of total variation in estimated effect sizes attributable to differences among the studies. An I^2^ value of 50% or higher is commonly considered an indicator of heterogeneity. As an additional measure of heterogeneity and to estimate the range of effect sizes in future studies, corresponding 95% prediction intervals will be calculated [50, 51].

For categorical data (yes/no), odds ratios (ORs) and 95% CIs will be estimated using a random-effects logistic regression model. Suicide-related outcomes, particularly suicide deaths, are expected to be rare (i.e. low counts). For rare events, the Mantel–Haenszel method will be used, which avoids continuity corrections that might bias results [52, 53]. To evaluate the robustness of the results, we will compare the results of the inverse variance model (which assumes a common treatment effect) and those of the Mantel–Haenszel method. If notable discrepancies arise between the methods, only the Mantel–Haenszel method will be used. Forest plots will be used for the visualization of the results, applying a 0.5 continuity correction for studies with zero events in one treatment arm. We will report the I^2^ statistic, along with its 95% CI, as an indicator of heterogeneity for all analyses. Prediction intervals will be calculated as an additional indicator for the degree of heterogeneity and to estimate the effect size range in future studies [50, 51].

All analyses will be conducted in R using the meta package [54]. Although we anticipate conducting meta-analyses for the primary and secondary outcomes, we will provide a systematic narrative synthesis if quantitative synthesis is not appropriate (e.g. due to insufficient data). Information will be presented in the text and in tabular form to summarize and explain the characteristics and findings of the included studies. The narrative synthesis will assess the relationships and results within and between the included studies.

### Subgroup analyses

Assuming that sufficient data are available, meta-regression will be used to explore potential subgroup differences based on study characteristics, including age group, psychiatric comorbidities, CBT-I delivery format (e.g. in-person vs. digital), treatment setting (e.g. outpatient vs. inpatient), and whether studies specifically recruited participants with suicidal ideation and behaviors or assessed these outcomes as a secondary endpoint.

### Sensitivity analyses

To assess the robustness of all synthesized results, we will conduct sensitivity analyses excluding studies at high overall risk of bias.

### Publication bias

Provided that ≥10 studies are included [55], the presence of potential publication bias (or “small-study effects”) will be assessed by examining asymmetry in a contour-enhanced funnel plot [56] using the primary outcomes.

### Risk of bias

Two reviewers will independently assess the risk of bias of the primary outcomes in each study using the Cochrane Risk of Bias 2 (RoB 2) tool [57]. The assessment will cover the following domains:

(1) Bias arising from the randomization process,

(2) Bias due to deviations from the intended interventions,

(3) Bias due to missing outcome data,

(4) Bias in measurement of the outcome and

(5) Bias in selection of the reported result.

Each domain will be rated as “low risk of bias”, “some concerns”, or “high risk of bias”. The overall risk of bias of each primary outcome will be determined by the least favorable rating among the domains. The assessments will be managed using the RoB 2 Excel tool. Disagreements will be resolved by consensus or, if necessary, with the involvement of another member of the review team.

### Certainty of evidence

Two reviewers will independently assess the certainty of the evidence for the primary outcomes using the Grading of Recommendations, Assessment, Development and Evaluation (GRADE) approach [58]. Based on GRADE guidelines [59], the following factors will be considered: (1) risk of bias (study limitations), (2) inconsistency of results (heterogeneity in the meta-analysis), (3) indirectness (relevance to the research question), (4) imprecision (small sample sizes, wide CIs), and (5) publication bias. The certainty in the body of evidence will be rated as high, moderate, low, or very low. Justifications will be provided for decisions to downgrade or upgrade the certainty of the evidence, as well as for the importance rating of each outcome. Disagreements will be resolved by consensus or, if necessary, with the involvement of another member of the review team.

## Discussion

The planned meta-analysis will offer a synthesis of the current evidence on the effects of CBT-I on suicide-related outcomes in individuals with insomnia. Given the strong and growing evidence linking insomnia with suicidal ideation and behaviors [5–10], the findings could have important implications for clinical practice and suicide prevention strategies. Notably, CBT-I in digitally delivered formats (digital CBT-I) offers a scalable, low-cost, and accessible alternative to face-to-face CBT-I [60], making it a promising component of broader public health approaches to suicide prevention. The planned meta-analysis may also play a critical role in identifying gaps in the literature, highlighting important methodological considerations, and informing the design of future studies.

Yet, it is important to acknowledge that RCTs in this specific area of research have only recently begun to emerge [27], and the current evidence base may still be limited in both size and scope. Another important consideration is that studies may differ in whether they recruited participants specifically for suicidal thoughts and behaviors (STBs) or measured STBs as a secondary outcome. These differences may influence the results, as the former may involve higher-risk populations with more severe psychiatric symptoms, while the latter may include less severe cases and thus be more prone to floor effects. To address this, we plan to conduct a meta-regression comparing these study types, provided that a sufficient number of studies are available. If not, this limitation will be clearly acknowledged in the interpretation of the results.

## Supplementary Material

CBT-I_suicideprev_Supplement_zpaf070

## References

[1] Morin CM, Jarrin DC. Epidemiology of insomnia: prevalence, course, risk factors, and public health burden. *Sleep Med Clin*. 2022;17(2):173–191. 10.1016/j.jsmc.2022.03.003 35659072

[2] Kyle SD, Morgan K, Espie CA. Insomnia and health-related quality of life. *Sleep Med Rev*. 2010;14(1):69–82. 10.1016/j.smrv.2009.07.004 19962922

[3] Chalet FX, Albanese E, Egea Santaolalla C, et al. Epidemiology and burden of chronic insomnia disorder in Europe: an analysis of the 2020 National Health and wellness survey. *J Med Econ*. 2024;27(1):1309–1320. 10.1080/13696998.2024.2407631 39318277

[4] Hertenstein E, Feige B, Gmeiner T, et al. Insomnia as a predictor of mental disorders: a systematic review and meta-analysis. *Sleep Med Rev*. 2019;43:96–105. 10.1016/j.smrv.2018.10.006 30537570

[5] Pigeon WR, Pinquart M, Conner K. Meta-analysis of sleep disturbance and suicidal thoughts and behaviors. *J Clin Psychiatry*. 2012;73(09):e1160–e1167. 10.4088/JCP.11r07586 23059158

[6] Bernert RA, Kim JS, Iwata NG, Perlis ML. Sleep disturbances as an evidence-based suicide risk factor. *Curr Psychiatry Rep*. 2015;17(3):554. 10.1007/s11920-015-0554-4 25698339 PMC6613558

[7] Baldini V, Gnazzo M, Rapelli G, et al. Association between sleep disturbances and suicidal behavior in adolescents: a systematic review and meta-analysis. *Front Psych*. 2024;15:1341686. 10.3389/fpsyt.2024.1341686 PMC11483864 39421072

[8] Liu RT, Steele SJ, Hamilton JL, et al. Sleep and suicide: a systematic review and meta-analysis of longitudinal studies. *Clin Psychol Rev*. 2020;81:101895. 10.1016/j.cpr.2020.101895 32801085 PMC7731893

[9] Malik S, Kanwar A, Sim LA, et al. The association between sleep disturbances and suicidal behaviors in patients with psychiatric diagnoses: a systematic review and meta-analysis. *Syst Rev.* 2014;3(1). 10.1186/2046-4053-3-18 PMC3945796 24568642

[10] Liu J-W, Tu Y-K, Lai Y-F, et al. Associations between sleep disturbances and suicidal ideation, plans, and attempts in adolescents: a systematic review and meta-analysis. *Sleep.* 2019;42(6). 10.1093/sleep/zsz054 30843072

[11] Riemann D, Espie CA, Altena E, et al. The European insomnia guideline: an update on the diagnosis and treatment of insomnia 2023. *J Sleep Res*. 2023;32(6):e14035. 10.1111/jsr.14035 38016484

[12] Hertenstein E, Trinca E, Wunderlin M, et al. Cognitive behavioral therapy for insomnia in patients with mental disorders and comorbid insomnia: a systematic review and meta-analysis. *Sleep Med Rev.* 2022;62:101597. 10.1016/j.smrv.2022.101597 35240417

[13] Türkmen C, Schneider CL, Viechtbauer W, et al. Cognitive behavioral therapy for insomnia across the spectrum of alcohol use disorder: a systematic review and meta-analysis. *Sleep Med Rev.* 2025;80:102049. 10.1016/j.smrv.2025.102049 39864131

[14] Furukawa Y, Nagaoka D, Sato S, et al. Cognitive behavioral therapy for insomnia to treat major depressive disorder with comorbid insomnia: a systematic review and meta-analysis. *J Affect Disord*. 2024;367:359–366. 10.1016/j.jad.2024.09.017 39242039

[15] Brower KJ . Insomnia, alcoholism and relapse. *Sleep Med Rev*. 2003;7(6):523–539. 10.1016/s1087-0792(03)90005-0 15018094

[16] Khurshid KA . Comorbid insomnia and psychiatric disorders: an update. *Innov Clin Neurosci*. 2018;15(3-4):28–32 PMC5906087 29707424

[17] Pigeon WR, Caine ED. Insomnia and the risk for suicide: does sleep medicine have interventions that can make a difference? *Sleep Med*. 2010;11(9):816–817. 10.1016/j.sleep.2010.06.002 20817603 PMC3106987

[18] Trockel M, Karlin BE, Taylor CB, Brown GK, Manber R. Effects of cognitive behavioral therapy for insomnia on suicidal ideation in veterans. *Sleep*. 2015;38(2):259–265. 10.5665/sleep.4410 25515115 PMC4288607

[19] Jernelöv S, Forsell E, Kaldo V, Blom K. Initial low levels of suicidal ideation still improve after cognitive Behavioral therapy for insomnia in regular psychiatric care. *Front Psych*. 2021;12:676962. 10.3389/fpsyt.2021.676962 PMC8273305 34262491

[20] Kalmbach DA, Cheng P, Ahmedani BK, et al. Cognitive-behavioral therapy for insomnia prevents and alleviates suicidal ideation: insomnia remission is a suicidolytic mechanism. *Sleep.* 2022;45(12). 10.1093/sleep/zsac251 PMC9742891 36242607

[21] Furukawa Y, Sakata M, Furukawa TA, Efthimiou O, Perlis M. Initial treatment choices for long-term remission of chronic insomnia disorder in adults: a systematic review and network meta-analysis. *Psychiatry Clin Neurosci*. 2024;78(11, 11):646–653. 10.1111/pcn.13730 39188094 PMC11804918

[22] McCall WV, Black CG. The link between suicide and insomnia: theoretical mechanisms. *Curr Psychiatry Rep*. 2013;15(9):389. 10.1007/s11920-013-0389-9 23949486 PMC3791319

[23] McCall WV, Looney SW, Zulfiqar M, et al. Daytime autonomic nervous system functions differ among adults with and without insomnia symptoms. *J Clin Sleep Med*. 2023;19(11):1885–1893. 10.5664/jcsm.10704 37421322 PMC10620659

[24] McCall WV, Sareddy S, Youssef NA, Miller BJ, Rosenquist PB. The pupillary light reflex as a point-of-care test for suicide risk: preliminary results. *Psychiatry Res*. 2021;295:113582. 10.1016/j.psychres.2020.113582 33234325

[25] Altena E, Ellis J, Camart N, Guichard K, Bastien C. Mechanisms of cognitive behavioural therapy for insomnia. *J Sleep Res*. 2023;32(6):e13860. 10.1111/jsr.13860 36866434

[26] Maurer LF, Espie CA, Kyle SD. How does sleep restriction therapy for insomnia work? A systematic review of mechanistic evidence and the introduction of the triple-R model. *Sleep Med Rev*. 2018;42:127–138. 10.1016/j.smrv.2018.07.005 30177248

[27] McCall WV . Targeting insomnia symptoms as a path to reduction of suicide risk: the role of cognitive behavioral therapy for insomnia (CBT-I). *Sleep.* 2022;45(12). 10.1093/sleep/zsac260 36306445

[28] Pigeon WR, Funderburk JS, Cross W, Bishop TM, Crean HF. Brief CBT for insomnia delivered in primary care to patients endorsing suicidal ideation: a proof-of-concept randomized clinical trial. *Transl*. *Behav Med*. 2019;9(6):1169–1177. 10.1093/tbm/ibz108 31271210

[29] Pigeon W, Crean H, Bishop T, Cross W, Youngren W, Funderburk J. 0634 an RCT of brief CBT for insomnia among primary care patients with suicidal ideation. *Sleep.* 2023;46(Supplement_1):A279. 10.1093/sleep/zsad077.0634

[30] Simon L, Steinmetz L, Feige B, Benz F, Spiegelhalder K, Baumeister H. Comparative efficacy of onsite, digital, and other settings for cognitive behavioral therapy for insomnia: a systematic review and network meta-analysis. *Sci Rep*. 2023;13(1):1929. 10.1038/s41598-023-28853-0 36732610 PMC9894949

[31] Mournet AM, Kleiman EM. A systematic review and meta-analysis on the efficacy of sleep interventions to treat suicidal ideation. *J Sleep Res*. 2024;33(4):e14133. 10.1111/jsr.14133 38164094

[32] Nazem S, Sun S, Barnes SM, et al. Impact of an internet-based insomnia intervention on suicidal ideation and associated correlates in veterans at elevated suicide risk. *Transl Behav Med*. 2024;14(11):673–683. 10.1093/tbm/ibae032 38864695 PMC11568844

[33] Crosby ES, Troop-Gordon W, Witte TK. A pilot randomized-controlled trial of sleep scholar: a brief, internet-based insomnia intervention for college students. *Journal article Behav*. 2024;56(2):366–380. 10.1016/j.beth.2024.06.007 40010906

[34] Nct . RCT of brief CBT-I in primary care veterans with suicidal thoughts Trial registry record. *https://clinicaltrialsgov/ct2/show/NCT03603717*. 2018;

[35] Moher D, Shamseer L, Clarke M, et al. Preferred reporting items for systematic review and meta-analysis protocols (PRISMA-P) 2015 statement. *Syst Rev*. 2015;4(1). 10.1186/2046-4053-4-1 PMC4320440 25554246

[36] Shamseer L, Moher D, Clarke M, et al. Preferred reporting items for systematic review and meta-analysis protocols (PRISMA-P) 2015: elaboration and explanation. *BMJ.* 2015;349(jan02 1):g7647. 10.1136/bmj.g7647 25555855

[37] Furukawa Y, Sakata M, Yamamoto R, et al. Components and delivery formats of cognitive Behavioral therapy for chronic insomnia in adults: a systematic review and component network meta-analysis. *JAMA Psychiatry*. 2024;81(4):357–365. 10.1001/jamapsychiatry.2023.5060 38231522 PMC10794978

[38] American Psychiatric Association . Diagnostic and Statistical Manual of Mental Disorders (4th ed., Text Revision). Washington, DC: American Psychiatric Publishing; 2000.

[39] American Psychiatric Association . Diagnostic and Statistical Manual of Mental Disorders (5th ed.). DSM-5. Arlington, VA: American Psychiatric Publishing; 2013, 10.1176/appi.books.9780890425596

[40] American Academy of Sleep Medicine . International Classification of Sleep Disorders (3rd ed.). Darien, IL: American Academy of Sleep Medicine; 2014.

[41] World Health Organization . ICD-10: International Classification of Diseases (10th Revision). 2nd ed. Geneva: World Health Organization; 2004.

[42] World Health Organization . ICD-11: International Classification of Diseases (11th revision). Geneva: World Health Organization; 2022.

[43] Park K, Suh S. Cognitive-Behavioral therapy for insomnia: a review of the treatment effects on suicide. *J Sleep Med*. 2017;14(2):47–54. 10.13078/jsm.17007

[44] Ribeiro JD, Huang X, Fox KR, Franklin JC. Depression and hopelessness as risk factors for suicide ideation, attempts and death: meta-analysis of longitudinal studies. *Br J Psychiatry*. 2018;212(5):279–286. 10.1192/bjp.2018.27 29587888

[45] Beck AT, Kovacs M, Weissman A. Assessment of suicidal intention: the scale for suicide ideation. *J Consult Clin Psychol*. 1979;47(2):343–352. 10.1037/0022-006X.47.2.343 469082

[46] Posner K, Brown GK, Stanley B, et al. The Columbia-suicide severity rating scale: initial validity and internal consistency findings from three multisite studies with adolescents and adults. *Am J Psychiatry*. 2011;168(12, 12):1266–1277. 10.1176/appi.ajp.2011.10111704 22193671 PMC3893686

[47] Morin CM, Belleville G, Bélanger L, Ivers H. The insomnia severity index: psychometric indicators to detect insomnia cases and evaluate treatment response. *Sleep.* 2011;34(5):601–608. 10.1093/sleep/34.5.601 21532953 PMC3079939

[48] *EndNote*. Clarivate; 2023.

[49] Borenstein M, Hedges LV, Higgins JP, Rothstein HR. A basic introduction to fixed-effect and random-effects models for meta-analysis. *Res Synth Methods*. 2010;1(2):97–111. 10.1002/jrsm.12 26061376

[50] Borenstein M, Higgins JP, Hedges LV, Rothstein HR. Basics of meta-analysis: I(2) is not an absolute measure of heterogeneity. *Res Synth Methods*. 2017;8(1):5–18. 10.1002/jrsm.1230 28058794

[51] IntHout J, Ioannidis JPA, Rovers MM, Goeman JJ. Plea for routinely presenting prediction intervals in meta-analysis. *BMJ Open*. 2016;6(7):e010247. 10.1136/bmjopen-2015-010247 PMC4947751 27406637

[52] Mantel N, Haenszel W. Statistical aspects of the analysis of data from retrospective studies of disease. *J Natl Cancer Inst*. 1959;22(4):719–748 13655060

[53] Efthimiou O . Practical guide to the meta-analysis of rare events. *Evidence Based Mental Health*. 2018;21(2):72–76. 10.1136/eb-2018-102911 29650528 PMC10270432

[54] Balduzzi S, Rücker G, Schwarzer G. How to perform a meta-analysis with R: a practical tutorial. *Evid Based Ment Health*. 2019;22(4):153–160. 10.1136/ebmental-2019-300117 31563865 PMC10231495

[55] Sterne JA, Sutton AJ, Ioannidis JP, et al. Recommendations for examining and interpreting funnel plot asymmetry in meta-analyses of randomised controlled trials. *BMJ.* 2011;343(jul22 1):d4002. 10.1136/bmj.d4002 21784880

[56] Peters JL, Sutton AJ, Jones DR, Abrams KR, Rushton L. Contour-enhanced meta-analysis funnel plots help distinguish publication bias from other causes of asymmetry. *J Clin Epidemiol*. 2008;61(10):991–996. 10.1016/j.jclinepi.2007.11.010 18538991

[57] Sterne JAC, Savović J, Page MJ, et al. RoB 2: a revised tool for assessing risk of bias in randomised trials. *BMJ.* 2019;366:l4898. 10.1136/bmj.l4898 31462531

[58] GRADEpro GDT . GRADEpro guideline development tool [software]. *McMaster University and Evidence Prime*. Available from https://www.gradepro.org/cite

[59] Guyatt GH, Oxman AD, Vist GE, et al. GRADE: an emerging consensus on rating quality of evidence and strength of recommendations. *BMJ*. 2008;336(7650):924–926. 10.1136/bmj.39489.470347.AD 18436948 PMC2335261

[60] Drake CL . The promise of digital CBT-I. *Sleep.* 2016;39(1):13–14. 10.5665/sleep.5304 26564140 PMC4678356

