# Supplementary material for: Cognitive behavioral therapy for insomnia as a suicide prevention strategy: a protocol for a systematic review and meta-analysis
Source: Sleep Adv. 2025 Oct 9;6(4):zpaf070. doi: 10.1093/sleepadvances/zpaf070 (PMC12640200; doi:10.1093/sleepadvances/zpaf070)
Supplement: CBT-I_suicideprev_Supplement_zpaf070 [file cbt-i_suicideprev_supplement_zpaf070.docx]

**Supplementary Materials**

**Cognitive behavioral therapy for insomnia as a suicide prevention strategy: a protocol for a systematic review and meta-analysis**

Cagdas Türkmen^1^, Carlotta L. Schneider^2^, Yuki Furukawa^3,4^, Jens H. van Dalfsen^5^, William V. McCall^6^, Wilfred R. Pigeon^7,8^, Andrew S. Tubbs^9^, Michael L. Perlis^10^, Dieter Riemann^11,12^, Kai Spiegelhalder^11^, Knut Langsrud^13^, Håvard Kallestad^13,14^, Elisabeth Hertenstein^2^

^1^Department of Addictive Behaviour and Addiction Medicine, Central Institute of Mental Health, Medical Faculty Mannheim, University of Heidelberg, Mannheim, Germany
^2^Faculty of Medicine, Department of Psychiatry, University of Geneva, Geneva, Switzerland
^3^Technical University of Munich, TUM School of Medicine and Health, Department of Psychiatry and Psychotherapy, Munich, Germany
^4^Department of Neuropsychiatry, University of Tokyo Hospital, Tokyo, Japan
^5^Department of Psychiatry, University Medical Center Groningen, Groningen, The Netherlands
^6^Department of Psychiatry and Health Behavior, Medical College of Georgia, Augusta University, Augusta, GA, USA
^7^Department of Psychiatry, University of Rochester Medical Center, Rochester, NY, USA
^8^Center of Excellence for Suicide Prevention, U.S. Department of Veterans Affairs, Canandaigua, NY, USA
^9^Department of Psychiatry, Washington University School of Medicine, St. Louis, MO, USA
^10^Behavioral Sleep Medicine Program, Department of Psychiatry, The School of Nursing, University of Pennsylvania, Philadelphia, PA, USA
^11^Department of Psychiatry and Psychotherapy, Medical Center – University of Freiburg, Faculty of Medicine, University of Freiburg, Germany
^12^Center for Basics in NeuroModulation (NeuroModulBasics), Faculty of Medicine, University of Freiburg, Freiburg, Germany
^13^Department of Mental Healthcare, St. Olavs University Hospital, Trondheim, Norway
^14^Department of Mental Health, Norwegian University of Science and Technology, Trondheim, Norway

**Corresponding author**Cagdas Türkmen
Central Institute of Mental Health, Department of Addictive Behaviour and Addiction Medicine
J5, 68159 Mannheim, Germany
Email: cagdas.tuerkmen@zi-mannheim.de
Phone: +49 621 1703-3533

**Supplement 1. PRISMA-P 2015 Checklist**

**For peer review reference.**

*Note.* This checklist was updated on 07/10/2025 following peer review and Editorial comments in *SLEEP Advances*. The line numbers have been revised to correspond to the clean version of the updated protocol (version 3, 07/10/2025) and do not correspond to those in the published article.

This checklist has been adapted for use with systematic review protocol submissions to BioMed Central journals from Table 3 in Moher D et al**:** Preferred reporting items for systematic review and meta-analysis protocols (PRISMA-P) 2015 statement. *Systematic Reviews* 2015 **4**:1

An Editorial from the Editors-in-Chief of *Systematic Reviews* details why this checklist was adapted - Moher D, Stewart L & Shekelle P**:** Implementing PRISMA-P: recommendations for prospective authors. *Systematic Reviews* 2016 **5**:15

| **Section/topic** | **#** | **Checklist item** | **Information reported** | | **Line number(s)** |
| --- | --- | --- | --- | --- | --- |
|  |  |  | **Yes** | **No** |  |
| **ADMINISTRATIVE INFORMATION** | | | | | |
| **Title** | | | | | |
| Identification | 1a | Identify the report as a protocol of a systematic review |  |  | 1-2 |
| Update | 1b | If the protocol is for an update of a previous systematic review, identify as such |  |  | N/A |
| **Registration** | 2 | If registered, provide the name of the registry (e.g., PROSPERO) and registration number in the Abstract |  |  | 66 |
| **Authors** | | | | | |
| Contact | 3a | Provide name, institutional affiliation, and e-mail address of all protocol authors; provide physical mailing address of corresponding author |  |  | 3-34 |
| Contributions | 3b | Describe contributions of protocol authors and identify the guarantor of the review |  |  | 303-305 |
| **Amendments** | 4 | If the protocol represents an amendment of a previously completed or published protocol, identify as such and list changes; otherwise, state plan for documenting important protocol amendments |  |  | 124-125 |
| **Support** | | | | | |
| Sources | 5a | Indicate sources of financial or other support for the review |  |  | 306-309 |
| Sponsor | 5b | Provide name for the review funder and/or sponsor |  |  | 301-302 |
| Role of sponsor/funder | 5c | Describe roles of funder(s), sponsor(s), and/or institution(s), if any, in developing the protocol |  |  | N/A |
| **INTRODUCTION** | | | | | |
| **Rationale** | 6 | Describe the rationale for the review in the context of what is already known |  |  | 75-116 |
| **Objectives** | 7 | Provide an explicit statement of the question(s) the review will address with reference to participants, interventions, comparators, and outcomes (PICO) |  |  | 117-119 |
| **METHODS** | | | | | |
| **Eligibility criteria** | 8 | Specify the study characteristics (e.g., PICO, study design, setting, time frame) and report characteristics (e.g., years considered, language, publication status) to be used as criteria for eligibility for the review |  |  | 126-139 |
| **Information sources** | 9 | Describe all intended information sources (e.g., electronic databases, contact with study authors, trial registers, or other grey literature sources) with planned dates of coverage |  |  | 140-148 |
| **Search strategy** | 10 | Present draft of search strategy to be used for at least one electronic database, including planned limits, such that it could be repeated |  |  | 143-147 Supplement 2 |
| ***STUDY RECORDS*** | | | | | |
| Data management | 11a | Describe the mechanism(s) that will be used to manage records and data throughout the review |  |  | 169-171 193-194 |
| Selection process | 11b | State the process that will be used for selecting studies (e.g., two independent reviewers) through each phase of the review (i.e., screening, eligibility, and inclusion in meta-analysis) |  |  | 172-184 |
| Data collection process | 11c | Describe planned method of extracting data from reports (e.g., piloting forms, done independently, in duplicate), any processes for obtaining and confirming data from investigators |  |  | 185-202 |
| **Data items** | 12 | List and define all variables for which data will be sought (e.g., PICO items, funding sources), any pre-planned data assumptions and simplifications |  |  | 187-191 194-199 |
| **Outcomes and prioritization** | 13 | List and define all outcomes for which data will be sought, including prioritization of main and additional outcomes, with rationale |  |  | 149-167 |
| **Risk of bias in individual studies** | 14 | Describe anticipated methods for assessing risk of bias of individual studies, including whether this will be done at the outcome or study level, or both; state how this information will be used in data synthesis |  |  | 250-261 244-245 |
| ***DATA*** | | | | | |
| **Synthesis** | 15a | Describe criteria under which study data will be quantitatively synthesized |  |  | 204-205 |
|  | 15b | If data are appropriate for quantitative synthesis, describe planned summary measures, methods of handling data, and methods of combining data from studies, including any planned exploration of consistency (e.g., *I* ^2^, Kendall’s tau) |  |  | 203-231 |
|  | 15c | Describe any proposed additional analyses (e.g., sensitivity or subgroup analyses, meta-regression) |  |  | 237-245 |
|  | 15d | If quantitative synthesis is not appropriate, describe the type of summary planned |  |  | 231-236 |
| **Meta-bias(es)** | 16 | Specify any planned assessment of meta-bias(es) (e.g., publication bias across studies, selective reporting within studies) |  |  | 246-249 |
| **Confidence in cumulative evidence** | 17 | Describe how the strength of the body of evidence will be assessed (e.g., GRADE) |  |  | 262-271 |

**Supplement 2. Search strings**

## Ovid Medline

### 21.3.25, 58

| (  Exp "suicide"/ OR  suicid*.ti,ab,kf.  )  AND |
| --- |
| (  Exp "Sleep Wake Disorders"/ OR  Insomnia*.ti,ab,kf. OR  sleep*.ti,ab,kf.  ) AND |
| (  Exp "Cognitive Behavioral Therapy"/ OR  CBTI.ti,ab,kf. OR  CBT.ti,ab,kf. OR  (cognitive ADJ3 (therap* OR psychotherap*)).ti,ab,kf. OR  ("cognitive behavio*").ti,ab,kf. OR  (behavioral ADJ3 (therap* OR intervention* OR treatment* OR measurement*)).ti,ab,kf.  ) |
| AND  (  Exp "Controlled Clinical Trial"/ OR  "clinical trials as topic"/ OR  "clinical trial*".ti,ab,kf. OR  random*.ti,ab,kf. OR  placebo.ti,ab,kf. OR  trial.ti.  ) |

## Embase

### 21.3.25, 208

| (  'suicidal behavior'/exp OR  suicid*:ti,ab,kw  )  AND |
| --- |
| (  'sleep disorder'/exp OR  Insomnia*:ti,ab,kw OR  sleep*:ti,ab,kw  )  AND |
| (  "Cognitive Behavioral Therapy"/exp OR  CBTI:ti,ab,kw OR  CBT:ti,ab,kw OR  (cognitive NEAR/3 (therap* OR psychotherap*)):ti,ab,kw OR  ("cognitive behavio*"):ti,ab,kw OR  (behavioral NEAR/3 (therap* OR intervention* OR treatment* OR measurement*)):ti,ab,kw  )  AND |
| ('controlled clinical trial'/exp OR 'clinical trial (topic)'/de OR 'clinical trial*':ti,ab,kw OR 'random*':ti,ab,kw OR 'placebo':ti,ab,kw OR 'trial':ti) |

## Cochrane Library

### 21.3.25, 220

| (  [mh "suicide"] OR  suicid*:ti,ab,kw  )  AND |
| --- |
| (  [mh "Sleep Wake Disorders"] OR  Insomnia*:ti,ab,kw OR  sleep*:ti,ab,kw  )  AND |
| (  [mh "Cognitive Behavioral Therapy"] OR  CBTI OR  CBT OR  (cognitive NEAR/3 *therap*) OR  (cognitive NEXT behavio*) OR  (behavioral NEAR/3 (therap* OR intervention* OR treatment* OR measurement*))  ):ti,ab,kw |

## PsycInfo (EBSCO)

### 21.3.25, 32

| (  suicid*  )  AND |
| --- |
| (  Insomnia* OR  sleep*  )  AND |
| (  CBTI OR  CBT OR  (cognitive N2 (therap* OR psychotherap*)) OR  "cognitive behavio*" OR  (behavioral N2 (therap* OR intervention* OR treatment* OR measurement*))  ) |
| AND  MR "clinical trial" |

## ClinicalTrials.gov

### 21.3.25, 29

Condition/disease:

(suicide OR suicidal OR suicidality) AND (insomnia OR sleep)

Intervention/treatment:

(cbt OR cbti OR cognitive OR behavioral)

## Deduplication

Partially automated deduplication in EndNote moved 128 duplicates to the Trash folder.
